# Olfactory function and discrimination ability in the elderly: a pilot study

**DOI:** 10.1186/s12576-022-00832-6

**Published:** 2022-04-01

**Authors:** Sae Uchida, Chiho Shimada, Naoko Sakuma, Fusako Kagitani, Akiko Kan, Shuichi Awata

**Affiliations:** 1 Department of Autonomic Neuroscience, Tokyo Metropolitan Institute of Gerontology, 35-2 Sakaecho, Itabashi-ku, Tokyo, 173-0015 Japan; 2 Department of Exploring End-of-Life Care for the Elderly, Tokyo Metropolitan Institute of Gerontology, Tokyo, Japan; 3 Department of Dementia and Mental Health Research, Tokyo Metropolitan Institute of Gerontology, Tokyo, Japan; 4 Department of Health Services Research, Tokyo Metropolitan Institute of Gerontology, Tokyo, Japan; 5 Integrated Research Initiative for Living Well With Dementia, Tokyo Metropolitan Institute of Gerontology, Tokyo, Japan; 6 Present Address: Faculty of Humanities and Social Science, Saku University, Nagano, Japan

**Keywords:** Odor identification threshold, Olfactory function, Cognitive function, Discrimination ability, Cholinergic system, Elderly people

## Abstract

We recently reported that subjects with a higher olfactory identification threshold for rose odor declined more in attentional ability in the elderly. This study focuses on discrimination ability and olfactory identification threshold in twelve elderly subjects living in a community (age: 80.9 ± 1.6). Olfactory function was assessed by the rose odor identification threshold. We assessed the discrimination ability by distinguishing 5 similar odor pairs. Our results showed that the subjects with a higher olfactory identification threshold (≥ 5) declined more in discrimination ability (14% ± 14%, *p* = 0.03) compared to those with a lower threshold (≤ 4) (averaged value set at 100%). As discrimination ability is related to the basal forebrain cholinergic system, our results suggest that olfactory impairment links to the decline in cognitive function relating the cholinergic system.

## Background

Olfactory function, especially olfactory identification ability, declines with age after 65 [1]. In addition to the age-related impairment, it is well established that olfactory function decline is among the earliest symptoms of neurodegenerative diseases, such as Alzheimer’s disease and Parkinson’s disease especially in the elderly [2–5]. Ability to identify odorants has been reported to decline in patients with mild cognitive impairment (MCI) and Alzheimer’s disease, which corresponds to developing cognitive impairment [6, 7]. To establish olfactory deficit as an early preclinical indicator for MCI and/or neurodegenerative diseases, such as Alzheimer’s disease, based on the olfactory impairment level, cognitive function decline should be evaluated in the elderly living in a community, using appropriate assessments.

Among the odorant items used for smell identification test, the rose odor has found to become harder to identify with the cognitive decline [7, 8]. Recently, our pilot study showed that olfactory impairment assessed by the rose odor identification threshold is linked to cognitive functional decline, especially that of attentional ability, in elderly people living in the community [9]. In addition to attention ability, discrimination ability also undergoes early impairment related to MCI and Alzheimer’s disease [10–12]. In basic animal studies using rats and mice, discrimination tasks using perceptually stimuli, such as visual discrimination task [13] and odor discrimination task [14, 15] are used as a cognitive test. Therefore, discrimination ability impairment is predicted to be related to olfactory function decline in the elderly.

In this study, we focused on the discrimination ability, as a cognitive function, and aimed to clarify the relationship between olfactory function and discrimination ability in the elderly.

## Methods

### Subjects

A total of 12 elderly people (10 females and 2 males), aged 70–90 years, living in the community, participated in this study. None of the subjects had been clinically diagnosed with dementia or a history of stroke, and none had nasal congestion or a runny nose on the day of examination either.

This study analyzed the discrimination ability, accessed in the same subjects on the same day as in the study reported by Uchida et al. [9].

The study was conducted under the declaration of Helsinki and approved by the Human Research Ethics Committee of the Tokyo Metropolitan Institute of Gerontology. Written informed consent was obtained from all the participants.

### Assessment of olfactory identification threshold

The olfactory ability was assessed by identifying the threshold for a rose odor using 2-phenylethyl alcohol (CAS no. 60-12-8; Tokyo Chemical Industry, Tokyo, Japan), a dominant odor compound in natural rose petals. The odorant concentration, dilution solvent, and methods for the assessment were all the same as described previously [9]. Briefly, a serial tenfold odorant dilution of eight steps, from 1 to 8 (low-to-high) concentrations, was prepared with a starting concentration of 631 mg/ml [16]. In this study, we analyzed the lowest concentration step at which the subjects correctly identified the odor. When the subjects had naming the odor difficultly, they were asked to identify the odor using a card with the correct odorant names written on it (rose flower, faint sweet, flower, or plants). The olfactory test took place in a quiet, well-ventilated room at temperature of 22–25 °C. A portable local ventilation equipment (SMST-DD-W-HD, Shonan Maruhachi S-Tech Co., Ltd., Kanagawa, Japan) was set close to the odorant bottles. Subjects were asked to avoid using perfume on the day of testing, and eating or drinking anything except for water 30-min before testing.

### Discrimination ability assessment

In this study, we assessed the discrimination ability, as a cognitive function. The discrimination ability was assessed by distinguishing between 5 similar odor pairs, 4 being enantiomer substances among them (limonene, carvone, α-pinene, and menthol), used in discrimination ability assessments based on differences in odor quality in earlier human studies [17, 18]. All enantiomer substances were purchased from Tokyo Chemical Industry (Tokyo, Japan). The substances were diluted to each testing concentration using propylene glycol (Tokyo Chemical Industry) as follows: 16.9 mg/ml for ( − )-limonene (CAS no. RN5989-54-8) and ( + )-limonene (CAS no. RN5989-27-5), 96.0 mg/ml for (R)-( − )-carvone (CAS no. RN6485-40-1) and (S)-( +)-carvone (CAS no. 2244–16-8), 86.0 mg/ml for (1S)-( −)-α-pinene (CAS no. RN7785-26-4) and (1R)-( +)-α-pinene (CAS no. RN7785-70-8), and 66.7 mg/ml for ( −)-menthol (CAS no. RN2216-51–5) and ( +)-menthol (CAS no. RN15356-60–2), as reported previously [17, 18]. The fifth pair represented familiar odors in daily life, soy sauce and Worcester sauce, used without any dilution. We choose soy sauce and Worcester sauce, because they are both brown color and it is difficult to distinguish them by appearance.

The subjects were presented with a 30 ml clear glass bottle containing a 200 μl-odorant solvent-soaked paper and were asked to sniff the bottle twice. By the forced-choice triangular test procedure, the subjects were asked to compare three bottles and identify the one containing the odd stimulus. The odd stimulus was always ( −) for all 4 enantiomeric odor pairs, and soy sauce for the familiar odors. The interval between the three odor pair bottles was 3 s. The interval between the different odor pairs was 30 s. After assessing the 5 odor pairs (1st round), the 2nd round of the same 5 odor pairs (with altered odd stimulus order) was assessed following a 1-min resting period.

The number of odor pairs correctly discriminated reproducibly in both rounds was counted as the score for discrimination ability. Hence, the scores in this task ranged from 0 (the lowest score) to 5 (the highest score). By the 2-round assessment, the chance level was minimized to 11%.

### Data analysis

The values were presented as the means ± SEM, unless stated otherwise. Data analysis was performed using Prism 5 (Graph-Pad Software Inc., San Diego, CA, USA). The relationship between the olfactory identification threshold and discrimination ability was analyzed by Spearman rank correlation coefficient. The Mann–Whitney test was used for the comparison of discrimination ability between two groups of different olfactory abilities (low- vs. high-threshold groups). A *p* value ≤ 0.05 was considered to be statistically significant.

## Results

**Fig. 1:**
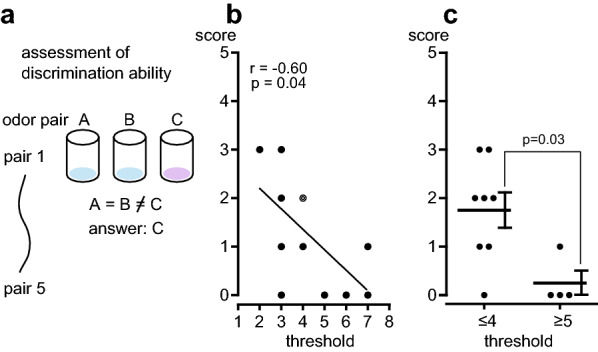
Relationship between discrimination ability and odor identification threshold. **a** Method testing the discrimination ability to distinguish between 5 odor pairs by comparing three bottles and identifying the one with an odd stimulus. **b** Scatter plot of the relationship between discrimination ability (score) and identification threshold for rose odor in all 12 subjects. The dots represent the values of an individual subject. The double circles (◎) represents overlapping two dots. Spearman's correlation coefficient *r* with *p* value. **c** Comparison of discrimination ability (score) between two groups of different olfactory abilities, that is, low- and high-threshold groups (≤ 4 and ≥ 5, respectively). The horizontal lines and vertical bars show the mean and SEM values in each group. *p* = 0.03; the significant difference between the low- and high-threshold groups, tested by Mann–Whitney test

As shown in Fig. 1a, the discrimination ability was assessed by forced-choice triangular test. All 12 subjects were able to detect the odorants used for the discrimination task. However, the ability to discriminate similar odors differed between subjects. The discrimination score of the 12 subjects was between 0 to 3. Figure 1b shows the scatter plot of the relationship between the discrimination (vertical axis, score) and olfactory (horizontal axis, threshold for identifying the rose odor) ability in all 12 subjects. All subjects were able to identify the odor between steps 2 and 7. The higher identification threshold showed a trend of lower discrimination score (*r* =  − 0.60, *p* = 0.04). In Fig. 1c, we compared the discrimination ability between the low- and the high-threshold groups (≤ 4; *n* = 8 and ≥ 5; *n* = 4, respectively). The discrimination score in the high-threshold group was 0.25 ± 0.25 (median: 0), and the value was significantly lower than that in the low-threshold group (1.8 ± 0.4, median: 2) (*p* = 0.03). When the discrimination score in the low-threshold group was set at 100% as an average, the score in the high-threshold group was calculated to be 14% ± 14%.

## Discussion

Our previous study showed that olfactory function decline (increase in identification threshold for rose odor) is linked to cognitive function decline, particularly that of attention ability, in elderly people living in the community [9]. In the present study, we showed the relationship between olfactory impairment and discrimination ability decline in the elderly.

As a common neural mechanism linking between olfactory dysfunction and cognitive decline in the elderly as well as in patients with neurodegenerative diseases, such as Alzheimer’s disease and Parkinson’s disease, possible contribution of basal forebrain cholinergic system had been suggested by the following studies. The cholinergic neurons in the basal forebrain project their fibers to the neocortex, hippocampus, and olfactory bulb, and has a key role in attention, memory, and olfactory function, respectively [19–21]. Our basic animal studies in rats and mice showed that the activation of the basal forebrain cholinergic neurons increases extracellular acetylcholine release and regulates regional blood flow in the neocortex, hippocampus, and olfactory bulb [22–28]. Research in human subjects showed that the cholinergic neurons in the basal forebrain undergo degeneration in the aging process, which is further increased by the presence of Alzheimer’s disease and Parkinson’s disease with dementia [29–31]. Recent publications revealed that basal forebrain atrophy precedes both entorhinal cortex atrophy and memory impairment in Alzheimer’s disease [32–34]. A positive association between olfactory identification ability and forebrain cholinergic pathway integrity has also been demonstrated [35].

In this study, discrimination ability was assessed by distinguishing between similar odor pairs (mainly enantiomer substances), according to previous studies [17, 18]. Animal experiments using rats and mice revealed that olfactory discrimination ability was increased by administration of physostigmine, an acetylcholine esterase inhibitor or by the optogenetic stimulation of basal forebrain cholinergic neurons projecting to the olfactory bulb [36, 37]. During the discrimination task, basal forebrain cholinergic neurons are recruited [38] and extracellular acetylcholine releases in the neocortex and hippocampus are increased [39, 40]. The aforementioned studies suggest the relation of basal forebrain cholinergic system in discriminative ability. Therefore, our present finding of linking olfactory impairment (increase in rose odor identification threshold) and discrimination ability decline could be related to impairment of basal forebrain cholinergic function.

In the present results, subjects with a higher olfactory threshold (≥ 5) declined more in the discrimination ability (14% ± 14%) compared with those subjects with a lower threshold (≤ 4) (averaged value was set at 100%). Our previous study investigated the relationship between olfactory identification threshold for rose odor and four cognitive measures [9]. Four cognitive measures were consisted of general cognitive ability assessed by Mini-Mental State Examination (MMSE) [41–43], its sub-domains (MMSE orientation and verbal recall 13-item subset, and 17 other items [7]), and attentional ability assessed by trail-making test part A (TMT-A) [44]. Attentional ability (performance speed of TMT-A), general cognitive ability (MMSE, total score), orientation and verbal recall (MMSE, 13-item subset), and other cognitive domains (MMSE, another 17-item subset) in the high-threshold group (≥ 5) were 73% ± 7%, 94% ± 6%, 94% ± 6%, and 95% ± 7%, respectively (when averaged value in the low-threshold group (≤ 4) was set at 100%, for each cognitive function). Considering together with our previous report [9], in the high-threshold group compared with the low-threshold group, the decline of the discrimination ability (14% ± 14%) was more marked than that of the attentional ability assessed by TMT-A (73% ± 7%) and other three cognitive measures. This may suggests that the discriminative ability assessed by distinguishing similar odor pairs sensitively measures cognitive impairment in MCI or even in earlier. Olfactory function assessment (identification threshold for rose odor) linked the discriminative ability might be useful for the early detection of Alzheimer’s disease, which could be applied to the elderly especially with mild cognitive impairment.

In this study, discriminative ability was assessed by the task distinguishing similar odor pairs. Certain previous studies reported discrimination ability decline in patients with MCI and Alzheimer’s disease, assessed by tactile angle discrimination [12] and visual object discrimination [10]. Future studies should clarify if the discrimination ability decline assessed using other sensory, such as tactile or vision, modalities could be also linked to olfactory impairment.

The limitation of this study is the small sample size. Therefore, the results should be interpreted with caution. Further studies with a larger sample size would be recommended to verify the link between olfactory identification ability and discriminative ability in the elderly. Moreover, the effects of possible confounding factors, such as age and education, should be clarified as well.

## Conclusions

This study shows that olfactory impairment assessed by rose odor identification threshold linked to the decline in cognitive function assessed by discrimination ability, in elderly people living in the community.

## Data Availability

The data that support the findings of this study are available from the corresponding author on reasonable request.

## Abbreviations

MCI: Mild cognitive impairment
MMSE: Mini-Mental State Examination
TMT-A: Trail-making test, part A
